# Drug-related problems among transfusion-dependent thalassemia patients: A real-world evidence study

**DOI:** 10.3389/fphar.2023.1128887

**Published:** 2023-04-20

**Authors:** Geok Ying Chun, Nurul Ain Mohd Tahir, Farida Islahudin, Veena Selvaratnam, Shu Chuen Li

**Affiliations:** ^1^ Faculty of Pharmacy, Universiti Kebangsaan Malaysia, Kuala Lumpur, Malaysia; ^2^ Centre for Clinical Trial, Ampang Hospital, Ampang, Selangor, Malaysia; ^3^ Hematology Department, Ampang Hospital, Ampang, Selangor, Malaysia; ^4^ School of Biomedical Sciences and Pharmacy, University of Newcastle, Callaghan, NSW, Australia

**Keywords:** drug-related problems, thalassemia, MRCI, DRP, risk factors

## Abstract

**Introduction:** Thalassemia is among the most common genetic disorders globally and many patients suffer from iron overload (IOL) complications that mainly affect the heart, liver and endocrine system. These events may be further complicated by drug-related problems (DRP), an inherent issue among patients with chronic diseases.

**Objective:** The study aimed to evaluate the burden, associated factors and impacts of DRP in transfusion-dependent thalassemia (TDT) patients.

**Method:** Eligible TDT patients under follow-up in a tertiary hospital between 01 March 2020 to 30 April 2021 were interviewed and their medical records were reviewed retrospectively to identify any DRP. DRPs were classified using the Pharmaceutical Care Network Europe (PCNE) classification version 9.1. The incidence and preventability of DRP were assessed and the associated risk factors were estimated by univariate and multivariate logistic regression.

**Results:** A total of 200 patients were enrolled with a median (interquartile range: IQR) age of 28 years at enrolment. Approximately 1 in 2 patients were observed to suffer from thalassemia-related complications. Throughout the study period, 308 DRPs were identified among 150 (75%) participants, with a median DRP per participant of 2.0 (IQR 1.0–3.0). Of the three DRP dimensions, treatment effectiveness was the most common DRP (55.8%) followed by treatment safety (39.6%) and other DRP (4.6%). The median serum ferritin level was statistically higher in patients with DRP compared with patients without DRP (3833.02 vs. 1104.98 μg/L, *p* < 0.001). Three risk factors were found to be significantly associated with the presence of DRP. Patients with frequent blood transfusion, moderate to high Medication Complexity Index (MRCI) and of Malay ethnicity were associated with higher odds of having a DRP (AOR 4.09, 95% CI: 1.83, 9.15; AOR 4.50, 95% CI: 1.89, 10.75; and AOR 3.26, 95% CI: 1.43, 7.43, respectively).

**Conclusion:** The prevalence of DRP was relatively high amongst TDT patients. Increased medication complexity, more severe form of the disease and Malay patients were more likely to experience DRP. Hence, more viable interventions targeted to these groups of patients should be undertaken to mitigate the risk of DRP and achieve better treatment outcomes.

## 1 Introduction

Drug-related problems (DRP) are commonly defined as events or circumstances involving drug therapy that actually or potentially interfere with the desired health outcomes (Pharmaceutical Care Network Europe Association, 2020). It is a public health problem that affects patients with chronic diseases (Niriayo et al., 2018; Garin et al., 2021; Ni et al., 2021). Studies in the past commonly focus on DRP of hospitalized patients, elderly population and patients with chronic diseases. These studies detected a relatively high median DRP prevalence of approximately 70% globally (Ni et al., 2021). At least half of the DRP identified were avoidable and when left unresolved, led to disease deterioration, unnecessary hospitalization and extra burden to the healthcare system (Zaman Huri and Chai Ling, 2013; Ni et al., 2021). Globally, DRP accounts for approximately USD 42 billion additional healthcare cost annually, which is a significant financial burden to the healthcare systems (Elliott et al., 2021; World Health Organization, 2022). Concerted efforts were initiated in tackling DRP with regulatory bodies putting greater emphasis on pharmacovigilance and the World Health Organization (WHO) mooting for a reduction in medication-related harm by 50% to facilitate efficient and sustainable healthcare (Donaldson et al., 2017). Additionally, studies in the past have revealed possible risk factors associated with DRP which serves as a cornerstone for preventive measures to tackle DRP. These risk factors include medication burden based on polypharmacy or a high number of daily doses, comorbidities and use of high-risk medications. These risk factors are also commonly observed in thalassemia patients (Niriayo et al., 2018; Ni et al., 2021), the patient group in our current study.

Thalassemia is a disease of deficiency or significant reduction in normal hemoglobin production leading to anemia. Patients with the more severe form of disease commonly require a lifelong regular blood transfusion, due to ineffective erythropoiesis. In many parts of the world, thalassemia is considered a rare disease, but it is undoubtedly the most common monogenic inherited disorder (Kattamis et al., 2020). Recent epidemiological data on thalassemia has shown an evolving shift in disease burden globally. Previously, thalassemia was predominantly found in tropical and subtropical regions, including the South East Asia region (Weatherall, 2011). However, due to global migration, the incidence of thalassemia is increasing in Europe and America (Kattamis et al., 2020). It is postulated that an estimated 5% of the global population are carriers of some form of hemoglobin defective gene and approximately 1.5% are *β* thalassemia carriers (Taher et al., 2018). It is expected that thalassemia will persist in countries in tropical and subtropical regions but the incidence may be somewhat moderated by proactive preventive measures such as premarital thalassemia screenings, genetic counselling and preimplantation genetic diagnosis (PGD) (Weatherall, 2018; Kattamis et al., 2020). Besides, with the increased lifespan due to improved pharmacotherapies, thalassemia is now considered as a chronic disease that imposes significant healthcare burden in countries with high prevalence. A recent study by Shafie et al., in 2018 estimated the lifetime cost of illness for each TDT patient in Malaysia to be USD 6,06,665, a significant financial burden to the local healthcare system as the cost is heavily subsidized by the Malaysian government.

Clinically, the management of thalassemia remains a challenge to most healthcare providers due to the sequelae of complications and limited treatment options. To date, the only curative option for this disease involves hematopoietic stem cell transplantation from a fully matched donor and the other would be gene therapy, both of which are not only costly but might lead to a greater risk of other therapy related complications (Khaddour et al., 2022). In order to reduce the risk of disease deterioration and further thalassemia complications in routine management, blood transfusions are recommended to ensure pretransfusion hemoglobin levels are sustained above 9 g/dL (Cappellini et al., 2021). However, regular transfusions would eventually lead to iron overload (IOL) with iron depositing mainly in the liver and the heart. These may in turn lead to complications including cardiomyopathy, liver cirrhosis and endocrine disorders and eventually death, if left untreated (Shah et al., 2019). Henceforth, lifelong administration of iron chelators is inevitable; but regular transfusion coupled with long-term chelation is burdensome to most thalassemia (Taher et al., 2018). The problem of treatment burden can lead to patient treatment fatigue, which has been associated with negative treatment behavior in other chronic diseases (Heckman et al., 2015). This includes poor adherence or use of complementary and alternative medicine as a substitute to control their complications which often results in treatment failure and increased risk of complications arising from harmful interactions (Heckman et al., 2015; Ismail et al., 2016).

Past studies had reported various drug-related problems in transfusion-dependent thalassemia (TDT) patients such as poor compliance, leading to difficulty to control iron load among these patients (Shah et al., 2019). This was especially pertinent among patients on deferoxamine (DFO) infusion where treatment compliance and QoL were affected by both prolonged painful infusions and the inconvenience of having frequent infusions (Porter et al., 2011). In an exploratory study by Babu et al. (2021) in the pediatric TDT population, a total of 16 DRP were found amongst 54 TDT patients and approximately half were attributed to failure to receive drugs and to a lesser extent subtherapeutic doses and adverse drug reactions. These findings may not be extrapolated to the adult population due to its small sample size and the population difference. Furthermore, adult patients tend to have other thalassemia related complications which may lead to increased treatment complexity and potentially give rise to other DRP (Taher et al., 2018). Nevertheless, globally, the incidence and patterns of DRP in adult TDT patients is not well documented and remains to be explored. Understanding the characteristics and contributory factors of DRP would provide new insight on potential means of handling such problems in the TDT population. This study aimed to determine the incidence and impact of DRP and its associated factors amongst TDT patients in the ambulatory setting.

## 2 Methods

### 2.1 Study design and settings

This was a cross sectional observational study of adult TDT patients in a tertiary public hospital, centrally located in the Klang Valley. It provides care for one-fourth of the registered thalassemia population in Malaysia. It is the main hematology referral center in Malaysia and serves as the main referral center for adult thalassemia patients in the Klang Valley and caters to patients in Peninsular Malaysia. As of end of 2018, it recorded the highest number of registered thalassemia patients under its care among the public hospitals in Malaysia (Mohd Ibrahim et al., 2020). It has a total number of 703 registered patients, which accounts for approximately 41% of the thalassemia patients in Klang Valley (Mohd Ibrahim et al., 2020).

### 2.2 Population

The minimal sample size of 150 patients was calculated using the Raosoft^©^ calculator. As there were no prior published DRP study on TDT patients, sample size estimation was based on a local ambulatory study by Zaman Huri and Chai Ling (2013) that reported a proportion of DRP of 0.9, with an absolute precision set at 0.05, confidence level of 95% and a 10% refusal rate. TDT patients were conveniently approached at the transfusion clinics between August 2021 and January 2022, screened to confirm their eligibility, and later invited to participate in the study after informed consent. Transfusion dependent thalassemia patients were defined as any thalassemia patients who required at least four regular transfusions annually (Mohd Ibrahim et al., 2020). TDT patients were enrolled if they were aged 18 years and above, had at least one clinic visit between March 2020 and April 2021 and were currently prescribed with at least one iron chelator agent. Participants with cognitive or mental incapacity or those with incomplete data, defined at 80% of missing data, were excluded from the study.

### 2.3 Data collection and recording

Clinical records of all enrolled participants between March 2020 and April 2021 were retrospectively extracted from the electronic Hospital Information System (eHIS) of the hospital. The relevant demographic, clinical and observational data were retrieved from the eHIS and collected using a standardized electronic data collection sheet on Research Electronic Data Capture (REDCap) by the investigator. The sociodemographic characteristics and latest clinical data including age, gender, ethnicity, history of drug allergy, splenectomy history, comorbidities, concomitant medications, and family history of thalassemia were obtained. Medication-related information were further quantified by medication count and classified into polypharmacy (use of five or more medication) (Masnoon et al., 2017). A validated patient-level MRCI tool was used to evaluate the complexity of medications regime (George et al., 2004). The MRCI tool evaluates medication complexity based on three aspects, namely, the medication dosage form, treatment frequency and any additional instructions. Patients were considered to have low medication complexity if the MRCI score was below 20, and moderate to high medication complexity if the MRCI score was 20 and above (Olson et al., 2014). Individuals’ laboratory readings for pretransfusion hemoglobin, serum ferritin, thyroid stimulating hormone, serum creatinine and liver transaminases over the one-year study period were retrieved and an average reading was tabulated. Thalassemia complications were recorded based on the presence of osteoporosis or osteopenia, cholelithiasis, diabetes mellitus, hypogonadism or amenorrhea, hypothyroidism or transfusion-related infections, as confirmed by the treating physician. As for the MRI T2* measured cardiac and liver iron load, it is commonly performed once every 2 years in Hospital as per local guidelines (Ministry of Health Malaysia, 2009). In the event that there was more than one measurement done in the preceding 24 months, the latest measurement was collected. Iron loading were considered present in the heart or liver with Cardiac T2*value of less than 20 ms or liver T2* value above 2 mg/g, respectively (Poggiali et al., 2012). Based on local laboratory reference range, the cardiac IOL was further categorized into mild (15–20 ms), moderate (10–15 ms), and severe (less than 10 ms) (Ministry of Health Malaysia, 2009) Similarly for Liver Iron Concentration (LIC), the liver IOL was categorized into mild (2–7 mg/g); moderate (7–15 mg/g) and severe (above 15 mg/g) (Ministry of Health Malaysia, 2009). The costs of medication were estimated based on the unit cost of medicine as of year 2021, provided by the procurement unit in the Pharmacy Department of hospital. The costs were expressed in both Malaysian Ringgit (MYR) and US dollars (USDs), as per 2021 exchange rate of US$ 1 = MYR 4.14 (World Bank, 2022).

### 2.4 Drug related problems classification and evaluation

Classification of DRP was done by a trained pharmacist in consultation with a haematologist using the PCNE tool (version 9.1). There are three primary domains of DRP and six subdomains (Pharmaceutical Care Network Europe Association, 2020). The potential DRP cases were identified from patients’ medical notes of the clinic visits during the study and cross-checked for any medication-related complaints, medication used against diagnosis, laboratory or radiography results. The Malaysian clinical practice guideline for thalassemia and drug product inserts were used to check for the appropriateness of drug used and dosage. Additionally, where possible during the transfusion visit, the participants were interviewed on any unpleasant experience or problems related to their thalassemia care, to identify additional DRP. Informed consent was obtained from each patient prior to the interviews.

Identified DRPs were then classified based on preventability using the Hepler Criteria to allow categorization of clinical outcomes of interest (Hepler and Strand, 1990). Clinical outcomes were identified based on the description of diagnosis by the treating physician. Drug information which included recommended dosages, side effects and potential drug interactions were obtained from Drug Information Handbook (Lexi-comp) and British National Formulary. The incidence of DRP and clinical outcomes were then analyzed.

### 2.5 Statistical analysis

Participants were categorized into groups with and without DRP. Descriptive statistics included median and interquartile range (IQR) for numerical variables and frequency and percentage for categorical variables. Normality of data was checked using the Kolmogorov-Smirnov test. The results showed that the dataset was skewed. Thus, non-parametric test was conducted using Mann-Whitney test to evaluate the difference in median serum ferritin levels and the median treatment cost annually between groups. Univariate and multivariate logistic regressions were used to evaluate factors associated with DRP. Variables with *p*-value ≤0.25 in univariate analysis were included in the multivariate logistic regression and covariates were included in the full model. Results of univariate and multivariate analyses were reported as odds ratio (OR) (Bursac et al., 2008). A *p*-value of <0.05 was considered statistically significant. All analyses were conducted using SPSS Statistics Version 28 (IBM Corporation, Software Group, Somers, NY, United States).

### 2.6 Ethical approval

Approval to conduct the study was obtained from Medical Research Ethics Committee at the Ministry of Health Malaysia (NMRR-20-2814-57656) and Research Ethics Committee of National University of Malaysia (UKM PPI/111/8/JEP-2022-231).

## 3 Results

### 3.1 Demographic characteristics of participants

A total of 208 patients were approached for the study. Of these, eight were excluded from the study as they were not diagnosed with TDT (N = 3), refused to participate (N = 2), non-thalassemia (N = 1), newly transferred from another hospital (N = 1) and medical record was not traceable (N = 1). A total of 200 eligible participants were enrolled into the study. Table 1 demonstrates the demographic and treatment characteristics of the 200 TDT patients enrolled. The median age of the TDT patient at enrolment was 28 years (IQR 24.0–36.0 years), 59% (*n* = 118) were female, and majority were Malays (*n* = 150, 75%). Most of the TDT patients were either beta-thalassemia major (*n* = 84, 42.0%) or HbE beta-thalassemia major (*n* = 87, 43.5%). Almost half (*n* = 98, 49%) of the study population were taking combination therapy of Deferiprone (DFP) and Deferoxamine (DFO) while 33% (*n* = 66) were on DFP only.

**TABLE 1:** Demographic and treatment characteristics of transfusion dependent thalassemia patients (*n* = 200).

Variable	Frequency (*n*, %)
Age(years), median (IQR)	28 (24.0–36.0)
Gender	
Male	82 (41.0)
Female	118 (59.0)
Ethnicity	
Malay	150 (75.0)
Chinese	43 (21.5)
Indian	2 (1.0)
Others	5 (2.5)
Thalassemia type	
Beta-thalassemia major	84 (42.0)
HbE beta-thalassemia major	87 (43.5)
HbH	16 (8.0)
Beta-thalassemia Intermedia	12 (6.0)
Others	1 (0.5)
Family history of thalassemia	
Yes	80 (40.0)
No	73 (36.5)
Unknown	47 (23.5)
History of drug allergy	
Yes	23 (11.5)
No	173 (86.5)
Unknown	4 (2.0)
Current iron chelation regimen	
S/C DFO	18 (9.0)
DFP	66 (33.0)
DFX	11 (5.5)
S/C DFO + DFP	98 (49.0)
Other combinations	7 (3.5)

Abbreviations: DFO, deferoxamine; DFP, deferiprone; DFX, deferasirox; s/c, subcutaneous.

### 3.2 Clinical characteristics of participants

A summary of clinical characteristics of the enrolled TDT samples is available at Table 2. Over one-third were on less than four weekly blood transfusions, but the average pretransfusion hemoglobin level of more than half of the patients (*n* = 131, 65.5%) was found to be lower than the recommended range of 9 g/dL. Meanwhile, 25.0% (*n* = 50) of the patients were splenectomized (which aims to ameliorate blood transfusion) at a mean age of 12.76 years (SD 7.64). Approximately half (*n* = 94, 47%) of the patients suffered from at least one of the common thalassemia-related complications. 37 (18.5%) patients had liver complications, of which 30 patients had hepatosplenomegaly, six had transaminitis (defined as value of Alanine Transaminase greater than three times the upper limit of normal), and one had liver cirrhosis secondary to iron overload. On the other hand, 13 (6.5%) had cardiovascular complications which included five pulmonary hypertension, two valvular heart disease, two cardiomyopathy, two heart failure and one case each of cardiomegaly, pericardial effusion and hypertension. On average, patients were taking six medications per day with median patient level MRCI score of 20.25 (IQR 13.25–26.00). Among the enrolled TDT patients, 111 had serum ferritin levels over 2,500 μg/L, indicating over half of the population (55.5%) had uncontrolled iron load. Based on the MRI T2* data, over 90% (*n* = 171) patients had some form of liver iron loading and approximately 1 in 4 had cardiac iron loading.

**TABLE 2:** Clinical characteristics of associated with transfusion dependent thalassemia participants (*n* = 200).

Characteristics	Frequency (*n*, %)^a^
History of splenectomy	
Yes	50 (25.0)
No	150 (75.0)
Transfusion frequency	
≤4 weekly	155 (77.5)
>4 weekly	45 (22.5)
Number of comorbidities per patient, median (IQR)	1 (0–2)
Number of daily doses, median (IQR)	8 (6–10)
Number of medications per day, median (IQR)	6 (4–7)
Polypharmacy	
Yes	146 (73.0)
No	54 (27.0)
MRCI Score, median (IQR)	20.25 (13.25–26.00)
≤20	96 (48.0)
>20	104 (52.0)
Serum ferritin level over past 12 months (μg/L), median (IQR)	3007.82 (1410.70–5209.40)
<2,500	89 (44.5)
≥2,500	111 (55.5)
Latest cardiac MRI T2*^b^(*n* = 185)	
No iron loading	140 (75.7)
Mild to moderate iron loading	18 (9.7)
Severe iron loading	27 (14.6)
Latest liver MRI T2*^c^ (*n* = 184)	
No iron loading	13 (7.1)
Mild to moderate iron loading	49 (26.6)
Severe Iron loading	122 (66.3)
Any thalassemia complications^d^	
Yes	94 (47.0)
No	106 (53.0)
Any liver complications	
Yes	37 (18.5)
No	163 (81.5)
Any cardiovascular complications	
Yes	13 (6.5)
No	187 (93.5)
Pretransfusion hemoglobin over past 12 months (g/dL), mean (SD)	8.43 (1.33)
<7	26 (13.0)
≥7 to <9	105 (52.5)
≥9	69 (34.5)
Average ALT (U/L) (*n* = 176), median (IQR)	40.00 (23.63–73.21)
Normal	103 (58.5)
Abnormal	73 (41.5)
TSH level over past 12 months (mIU/L) (*n* = 159), median (IQR)	2.43 (1.51–3.60)
Normal	137 (86.2)
Abnormal	22 (13.8)
Serum creatinine level over past 12 months (μmol/L) (*n* = 172), median (IQR)	49.00 (42.00–57.34)
Presence of any drug related problem	
Yes	150 (75.0)
No	50 (25.0)

^a^ Unless otherwise indicated, all results are presented as frequency (*n*, %).

^b^ No Cardiac iron loading is defined as MRI T2* value of above 20 ms. Mild to moderate cardiac iron loading is defined as MRI T2* of 10–20 ms. Severe cardiac iron loading is defined as MRI T2* of less than 10 ms

^c^ No Liver iron loading is defined as MRI T2* level of less than 2 mg/g. Mild to moderate liver iron loading is defined as MRI T2* of 2–15 mg/g. Severe liver iron loading is defined as MRI T2* of more than 15 mg/g.

^d^ Thalassemia complications defined as presence of any osteoporosis or osteopenia, cholelithiasis, diabetes mellitus, hypogonadism or amenorrhea, hypothyroidism or transfusion related infections.

Abbreviations: ALT, alanine transferase; IQR, interquartile range; MRI, magnetic resonance imaging; MRCI, medication regimen complexity index; SD, standard deviation; TSH, thyroid stimulating hormone.

### 3.3 Drug-related problems

Table 3 summarizes the type of DRP detected in the study. A total of 308 DRP were identified in 150 (75%) participants. The mean (±SD) DRP per participant was 2.10 ± 1.23 (range 1–6). From the DRP, 253 (82.1%) were evaluated to be preventable. There were 3 identified DRP categories whereby the most common was treatment effectiveness problems (*n* = 172, 55.8%), followed by treatment safety problems (*n* = 122, 39.6%) and the least common was other DRP (*n* = 14, 4.6%).

**TABLE 3:** Summary breakdown of drug related problems encountered (*n* = 308).

Classification of drug-related problems	*n* (%)
Preventability of drug related problem	
Yes	253 (82.1)
No	55 (17.9)
Treatment Effectiveness	172 (55.8)
Effect of drug treatment not optimal	136 (43.5)
No effect of drug treatment	19 (6.2)
Untreated symptoms or indication	19 (6.2)
Treatment safety: adverse drug event (possibly occurring)	122 (39.6)
DFO related local site reaction (pain, swelling, itch)	44 (35.8)
DFP related GI disturbance (nausea, vomiting, diarrhoea)	37 (30.1)
DFP related somnolence	11 (8.9)
DFP related arthralgia or myalgia	9 (7.3)
DFP/DFO related headache	6 (4.9)
Other ADRs	15 (12.3)
Others	14 (4.6)
Unclear problem or complaint (specified below)	
Administration problem	3 (1.0)
Lack monitoring	8 (2.6)
Wrong dose	1 (0.3)
Unnecessary drug	2 (0.7)

Abbreviations: DFO, deferoxamine; DFP, deferiprone; ADR, adverse drug reactions; GI, gastrointestinal.

From the treatment effectiveness problems category, 136 (43.5%) patients admittedly reduced or skipped treatment doses leading to a non-optimal treatment effect. The problems pertaining to treatment safety (*n* = 122, 39.6%) included gastrointestinal disturbances and somnolence associated with DFP (*n* = 48, 39%), local infusion site reactions such as pain and itchiness due to DFO (*n* = 44, 35.8%). Most of the side effects were mild in nature as no pharmacological intervention was required (*n* = 119, 97.5%).

### 3.4 Drug related problems and its associated clinical outcomes

Patients with DRP were associated positively with higher iron loading. Based on the average serum ferritin levels, 71.3% (*n* = 107) patients with DRP had inadequate chelation as indicated by ferritin level above 2,500 ug/L compared to only 8% (*n* = 4) in those without DRP. The Mann-Whitney U test showed that patients with DRP had significantly higher median serum ferritin level (3833.02 μg/L) than those without DRP (1104.98 μg/L) (*U* = 6591.50, *z* = 8.017, *p*-value<0.001). Similarly, there was association between DRP and both cardiac [Ⅹ^2^ (2) = 13.899, *p* < 0.001] and liver load [Ⅹ^2^ (2) = 24.48, *p* < 0.001] measured via MRI T2*(Table 4). A significantly higher proportion of patients with DRP had mild to severe iron load in the liver (*n* = 136, 79.5%) and the heart (*n* = 44, 97.7%) compared to those without DRP.

**TABLE 4:** Association between cardiac and liver MRI results with DRP status (*n* = 200).

Variable	*n*	DRP freq (%)	No DRP freq (%)	Ⅹ^2^ statistic (df)	*p-*value
Liver iron load					
Normal	13	7 (53.8)	6 (46.2)	24.48 (2)	<0.001
Mild to moderate	49	28 (57.1)	21 (42.9)		
Severe	122	108 (88.5)	14 (11.5)		
Cardiac iron load					
Normal	140	100 (71.4)	40 (28.6)	13.899 (2)	<0.001
Mild to moderate	18	17 (94.4)	1 (5.6)		
Severe	27	27 (100)	0 (0.0)		

Chi-square tests were performed.

Abbreviation: *n*, number; DRP, drug-related problems; freq, frequency.

The annual cost of all medications used in treatment of TDT patients with DRP (Median = MYR 19,366.63, IQR: 7175.64–26,684.76; US$ 4677.93, IQR: 1733.25–6555.59) was statistically significantly higher than those without DRP (Median = MYR 3682.32, IQR: 2631.01–24,025.56; US$ 889.45, IQR 635.51–5803.28) (*U = 4795.5, z =* 3.038*, p =* 0.002). There were nevertheless only 28 (14%) cases of additional hospital visits, all of which were attributed to the DRP group except one. The additional hospital visits included hospitalization (*n* = 10; 5%), emergency department visits (*n* = 8, 4%) or additional clinic visits (*n* = 21, 10.5%). Of these additional hospital visits, 2 cases of death were reported and both were due to cardiac complications secondary to IOL.

### 3.5 Factors associated with DRP

For univariate analysis, patient’s age, presence of specific comorbidities like diabetes mellitus and history of allergy that were previously found to be associated with DRP were excluded because they were observed to be statistically insignificant. Table 5 depicts the factors associated with DRP in our study including gender, ethnicity, presence of polypharmacy, average pretransfusion hemoglobin level, presence of multiple comorbidities, MRCI, and blood transfusion frequency had *p* values ≤0.25 in the univariate analysis. These factors were analyzed in the final model. Only three variables were statistically significant which included Malay ethnicity [Adjusted Odds ratio (AOR) 3.26; 95% confidence interval (CI): 1.43, 7.43], transfusion frequency of ≤4 weekly (AOR 4.09; 95% CI, 1.83, 9.15) and those with moderate to high medicine regimen complexity indices (AOR 4.50; 95% CI: 1.89, 10.75). Patients who were on more frequent transfusion visits (≤4 weekly) were 4.1 times more likely to experience any DRP compared to those who transfuse less frequently. Similarly, patients with moderate to high Medication Regimen Complexity Index (MRCI) (>20) had 4.5 times higher likelihood of experiencing at least 1 DRP. Malay TDT patients were 3.3 times more likely to experience any DRP compared to non-Malays while both polypharmacy and gender did not affect DRP occurrence.

**TABLE 5:** Factors associated with DRP among ambulatory TDT patients in Ampang Hospital (*n* = 200).

Factors	Univariate		Multivariate	
	Unadjusted OR (95% CI)	*p*-value	Adjusted OR (95% CI)	*p*-value
Gender				
Female	1	(Reference)	—	—
Male	0.848 (0.444–1.620)		—	—
Ethnic				
Non-Malay	1	(Reference)	3.258 (1.429–7.426)	0.005
Malay	1.826 (0.906–3.682)			
Polypharmacy				
No	1	(Reference)	1.045(0.448–2.438)	0.919
Yes	2.567* (1.299–5.076)			
Pretransfusion hemoglobin level (g/dL)				
≥9	1	(Reference)	—	—
<9	1.092(0.559–2.132)			
Multiple comorbidities				
<3 diseases	1	(Reference)	—	—
≥3 diseases	0.924 (0.425–2.009)			
Medication regimen complexity index (MRCI)				
≤20	1		4.503 (1.886–10.751)	<0.001
>20	4.390 (2.154–8.945)			
Transfusion frequency				
>4 weekly	1	Reference	4.089 (1.827–9.149)	<0.001
≤4 weekly	3.333 (1.638–6.782)			

Multiple logistic regression was adjusted for gender, pretransfusion hemoglobin level and multiple comorbidities. *p*-value significant at <0.05. The variance inflation factors (VIFs) for all variables were less than 10 and no interaction term were found. Model fit was examined using Hosmer-Lemeshow test (*p* = 0.156), classification table (80.0%), and area under the receiver operating characteristic curve (75.5%).

Abbreviation: OR, odds ratio; CI, confidence interval.

## 4 Discussion

To our knowledge, this is the first study that provides data on burden of DRP in patients with TDT hereditary disease in Malaysia. Understanding the prevalence of DRP and factors associated with DRP in TDT patients would be useful for relevant stakeholders to improve the efficiency of healthcare service delivery. In our study, we used a validated DRP tool to systematically identify and evaluate the DRP in an outpatient setting of a tax-subsidised public hospital. Despite being a single-centre study, Ampang Hospital receives hematology referrals from the most populous capital of Malaysia and its surrounding region. The patient characteristics from these regions are consistent with the Malaysian TDT population and therefore the results of our study could at least provide some reference information to other local settings (Mohd Ibrahim et al., 2020). In our study, we found that high proportion of TDT patients were affected by DRP even though the patients were mainly young adults, highlighting the need to address this issue. DRP were also found significantly attributable to the Malay ethnicity, moderate to high medication regimen complexity and frequent transfusion visits. These findings provide compelling new evidence to clinicians to risk stratify patients who are at risk of DRP as a form of future preventive measures.

Our study also showed a high prevalence of DRP, with approximately three-quarters of the studied TDT population being affected by DRP with an average of two DRP per patient. This is comparable to past review papers that reported DRP prevalence of 70% among patients in ambulatory setting (Ayele and Tesfaye, 2021; Ni et al., 2021). The magnitude of this problem was also comparable to the high incidence of DRP among hospitalised patients that ranged between 71.5% and 81.0%, therefore suggesting a need to look into DRP in the TDT population (Blix et al., 2004; Albayrak et al., 2022). However, the average DRP rate was lower compared to a hematology-oncology study in a hospital setting (Modesto et al., 2020). This may be due to the difference inpatient population in referenced study which involved severely ill hematology-oncology patients requiring more complex treatments with more toxic side effect profiles. In contrast, our study was conducted in the outpatient setting which involved more stable patients and the pharmacotherapy used in TDT patients were comparatively more tolerable with lesser adverse events than those for oncology patients. Hence; it was expected that the DRP incidence to be lower (Grech et al., 2021). In our study, treatment effectiveness was the predominant DRP observed with “treatment effect not optimal” being the top DRP subdomain identified. This is in congruent with the review by Garin et al., in 2021. Furthermore, most patients in our study were observed to have an appropriate iron chelation regimen doses optimized based on the body weight of patients, but the IOL problem in TDT patients remained unresolved as reflected by the suboptimal control in serum ferritin level among 55% of the patients. We believe that the suboptimal treatment effect was likely due to patient non-adherence, as most patients were on DFO which has long been associated with poor adherence (Porter, 2001; Mohd Ibrahim et al., 2020; Mohamed et al., 2022). Nevertheless, as the cause of DRP was not evaluated in our study, further studies are required to confirm this finding.

In line with previous studies, the presence of DRP was associated with poorer disease control (Zaman Huri and Chai Ling, 2013; Ni et al., 2021). This was evidenced by the presence of DRP which is positively associated with a higher incidence of IOL in the liver or heart among TDT patients, as reflected by the markedly deranged serum ferritin and MRI T2* levels. This persistently excess iron would potentially deposit mainly in the liver, contributing to liver cirrhosis and fibrosis, cardiac complications and eventual death (Krittayaphong et al., 2018; Taher et al., 2018). Additionally, the cost of medication among patients with DRP was approximately five times higher than those without DRP. This observation was unsurprising as the DRP was found to be associated with suboptimal control of IOL. This phenomenon leads to increased doses or addition of ICT as well as other medication for IOL related complications. Nevertheless, despite high incidence of DRP, the incidence of additional hospitalisations or emergency visits were relatively low at 8.7% compared to an average of 15.4% in another study (Ayalew et al., 2019). There might be several explanations to this finding. Firstly, our study was conducted in an outpatient setting where these chronically ill patients were relatively stable. As our study patients had been on treatment for some time, any DRP would be less severe and more tolerable in nature, hence not requiring hospitalisation. Besides, it is known that iron accumulates in the TDT patients silently over years and leads to IOL complications over time, therefore the effect of “treatment effect not optimal” may currently have minimal impact on patients’ life compared to later in life (Shah et al., 2022). In keeping with past studies done in similar region, our study found that the incidence of cardiac failure (1%) and liver cirrhosis (0.5%) were low despite significant number of patients with IOL (Lam et al., 2021). As explained previously by Taher and Cappellini (2018), aging increases the risk for patients to be affected by severe forms of cardiac and liver complications including cirrhosis and hepatocellular carcinoma as well as cardiac arrythmia and this natural disease progression would be expedited with the presence of IOL. Hence, continuous monitoring of clinical outcome and detection and control of DRP risk factors will be essential to avoid potential complications secondary to IOL.

In this study, medication regimen complexity was found to be one of the major risk factors for occurrence of DRP. Patients with moderate to high MRCI score were at significantly greater odds of having any DRP. This was consistent with the finding by Ferreira et al. (2015) where all patients in the primary care setting with higher MRCI (≥25) were found to be affected by at least one DRP. The median MRCI score was also comparable to that of other chronic diseases including diabetes and HIV in (Libby et al., 2013). Complex medication regimen in TDT patients were expected as these patients were commonly affected by thalassemia related complications that necessitate coadministration of multiple medication with complex dosing regimen. On the other hand, our data showed that polypharmacy, which was a significant predictor in many studies, did not significantly contribute to DRP in TDT population (Ma et al., 2019; Garin et al., 2021; Ni et al., 2021). These results demonstrated the advantage of evaluation of medication burden based on medication complexity over solely based on number of medications, concurring to the findings by Mansur et al. (2012). Our results highlighted the potential adoption of MRCI tool to facilitate risk stratification of TDT patients at risk of DRP to simplify the complex regimen and improve clinical and economic outcomes.

Another significant risk factor for DRP is ethnicity. Interestingly, our study found the Malay patients; the predominant ethnic group in Malaysia, to be more susceptible to DRP than those of non-Malay ethnicity. This was contrary to many past studies that highlighted ethnic minorities to be more susceptible to adverse medication effect and DRP, attributable to religious beliefs, knowledge and even health inequity (Alhomoud et al., 2013; Horne et al., 2013). The variation in our study may be explained by the disparity in patients’ attitude and belief towards illness and treatment. This may include fatalistic attitude towards their illness that has been previously documented as a potential cause of poor adherence and may also be the reason behind high DRP (Saidi et al., 2018; Chong et al., 2021). Intriguingly, there is a culture of using alternative medicine as naturally available products were perceived to be more effective and safer for consumption. Tellingly, this local culture may contribute towards the higher incidence of DRP (Ismail et al., 2005). Unfortunately, since we did not explore the reasons for DRP, we can only speculate that the ethnicity variations in DRP occurrence were due to the cultural and belief reasons, and therefore this observation would warrant further evaluation.

The likelihood of DRP significantly increase with disease severity, as inferred from the frequency of blood transfusion. Patients who were affected by the more severe form of thalassemia, were found to be at greater risk of DRP. Similar results were previously observed in hospitalized patients and also in other chronic diseases (Njeri et al., 2018; Garin et al., 2021). As patients transfuse more, they would have greater risk of IOL and its related complications and management may become more complex. These may have contributed to higher incidences of DRP.

There were several limiting factors in this study. Firstly, the study was conducted in a single-center using non-probability sampling method, due to logistic challenges in conducting the study especially during the Coronavirus disease 2019 (COVID-19 pandemic). Nevertheless, we managed to approach the TDT patients in the main adult thalassemia referral center which has the highest number of registered thalassemia patients and the demographic make-up was similar to the TDT population. Furthermore, the incidence of DRP may have been underestimated as the information was partly based on self-reporting by the participants. However, the impact was minimized as retrospective review of medical records was performed to identify other DRP. Last but not least, the study was conducted during the COVID-19 pandemic where there may possibly be disruptions in medication and blood supplies, as well as increase demand for medical care (Yu et al., 2021). These global disruption may have been confounded and led to more DRP being detected. Despite the limitations, our study shed some new light on the extent of DRP in rare diseases like thalassemia, which have been overlooked in the past. This study found that the incidence and types of DRP faced in these populations were similar to that of non-communicable diseases, which in turn led to poorer disease control. Furthermore, the potential risk factors associated with DRP identified in our study would allow the relevant stakeholders to strategically tackle and prevent the occurrence of DRP in this patient population globally. In particular, one of the possibly modifiable risk factors includes the moderate to high medication complexity which can potentially burden both the patients and their caretakers. This may be tackled by the possibility of built-in evaluation of DRP into pharmaceutical care with ongoing medication review of patients’ treatment. Future studies looking into the means and clinical implication of simplified medication regimen would be beneficial.

## 5 Conclusion

In conclusion, almost three-quarters of the TDT samples enrolled in this study had DRP resulting in poor thalassemia control. The findings highlight an urgent need to undertake measures, including medication reviews and counselling to reduce DRP. Our study also highlights several risk factors such as Malay ethnicity, more severe disease and moderate to high MRCI predisposing TDT patients to DRP. The gathered risk factors may facilitate identification of patients at risk of DRP and allow a more targeted approach in prevention of DRP.

## Data Availability

The original contributions presented in the study are included in the article/supplementary material, further inquiries can be directed to the corresponding author.
