# Corrigendum: Drug-related problems among transfusion-dependent thalassemia patients: a real-world evidence study

**DOI:** 10.3389/fphar.2023.1336072

**Published:** 2023-11-22

**Authors:** Geok Ying Chun, Nurul Ain Mohd Tahir, Farida Islahudin, Veena Selvaratnam, Shu Chuen Li

**Affiliations:** ^1^ Faculty of Pharmacy, Universiti Kebangsaan Malaysia, Kuala Lumpur, Malaysia; ^2^ Centre for Clinical Trial, Ampang Hospital, Ampang, Selangor, Malaysia; ^3^ Hematology Department, Ampang Hospital, Ampang, Selangor, Malaysia; ^4^ School of Biomedical Sciences and Pharmacy, University of Newcastle, Callaghan, NSW, Australia

**Keywords:** drug-related problems, thalassemia, MRCI, DRP, risk factors

In the published article, there was an error in the **Funding** statement. The name of the funder is missing (Ministry of Higher Education, Malaysia) and details of another funder is missing. The correct **Funding** statement appears below.

“The study acknowledges the Fundamental Research Grants Scheme (FRGS) [FRGS/1/2020/SS02/UKM/02/5] funded by the Ministry of Higher Education (MOHE), Malaysia, and part of this research is supported by the Geran Universiti Penyelidikan (GUP) funded by the Universiti Kebangsaan Malaysia, grant number GUP-2020-003”.

The authors apologize for this error and state that this does not change the scientific conclusions of the article in any way. The original article has been updated.

